# An Automated Skill Assessment Framework Based on Visual Motion Signals and a Deep Neural Network in Robot-Assisted Minimally Invasive Surgery

**DOI:** 10.3390/s23094496

**Published:** 2023-05-05

**Authors:** Mingzhang Pan, Shuo Wang, Jingao Li, Jing Li, Xiuze Yang, Ke Liang

**Affiliations:** 1 College of Mechanical Engineering, Guangxi University, Nanning 530004, Chinalijingao39@gmail.com (J.L.);; 2 State Key Laboratory for Conservation and Utilization of Subtropical Agro-Bioresources, Nanning 530004, China; 3 Guangxi Key Laboratory of Manufacturing System & Advanced Manufacturing Technology, School of Mechanical Engineering, Guangxi University, Nanning 530004, China

**Keywords:** robot-assisted minimally invasive surgery, surgical skill assessment, visual motion tracking, kernel correlation filter, residual neural network

## Abstract

Surgical skill assessment can quantify the quality of the surgical operation via the motion state of the surgical instrument tip (SIT), which is considered one of the effective primary means by which to improve the accuracy of surgical operation. Traditional methods have displayed promising results in skill assessment. However, this success is predicated on the SIT sensors, making these approaches impractical when employing the minimally invasive surgical robot with such a tiny end size. To address the assessment issue regarding the operation quality of robot-assisted minimally invasive surgery (RAMIS), this paper proposes a new automatic framework for assessing surgical skills based on visual motion tracking and deep learning. The new method innovatively combines vision and kinematics. The kernel correlation filter (KCF) is introduced in order to obtain the key motion signals of the SIT and classify them by using the residual neural network (ResNet), realizing automated skill assessment in RAMIS. To verify its effectiveness and accuracy, the proposed method is applied to the public minimally invasive surgical robot dataset, the JIGSAWS. The results show that the method based on visual motion tracking technology and a deep neural network model can effectively and accurately assess the skill of robot-assisted surgery in near real-time. In a fairly short computational processing time of 3 to 5 s, the average accuracy of the assessment method is 92.04% and 84.80% in distinguishing two and three skill levels. This study makes an important contribution to the safe and high-quality development of RAMIS.

## 1. Introduction

Recent years have witnessed the remarkable progress of RAMIS in general surgery, gastrointestinal surgery, urology, and gynecology due to the advantages of 3D vision, motion scaling, and tremor filtering [1,2]. RAMIS is a teleoperation mode based on the human–computer interaction system. As the most important link in the “doctor–robot–patient” system, the doctor’s operating skill level directly affects the operating effect of the entire surgical robot system, and it plays a decisive role in the safety and efficacy of the surgical procedure [3]. The effectiveness of these operations depends on the surgeon’s ability, which has a big impact on the patient’s health and safety [4,5]. In addition, a reliable method for assessing skills in RAMIS is essential in order to improve physicians’ technical skills [6]. The purpose of skill assessment is to help and guide medical staff to conduct more efficient and accurate skill training through the most reliable assessment means possible during the surgical training of medical staff. Therefore, before performing surgery, surgeons must acquire the necessary surgical operation skills. Accurate skill assessment techniques are essential for improving surgical abilities [7]. Therefore, identifying the skill level of robotic operations effectively in order to provide doctors with fair and objective theoretical guidance plays a crucial role in the research and improvement of robot system control methods, thus assisting medical personnel to enhance operational skills, and ensuring the standardization and safety of operations.

The majority of studies focus on assessing the motion signals of the SIT. Farcas et al. [8] used a traditional laparoscopic box trainer to install a customized motion tracking system in order to analyze and study the instrument motion at the stage of a suture task in vivo, as determined in the simulator. It provided an assessment of velocity and acceleration. One purpose of these simulators is to reduce the subjective reliance on experts and observers when evaluating performance or technical skills [9]. The key motion signals of the SIT have provided objective and precise evaluations of skill in surgical skill training [10]. Therefore, obtaining the key motion signals of the SIT has important research relevance. Jiang et al. [11] analyzed the key motion features, such as the SIT’s trajectory, and distinguished the motion control skills of operators with different skill levels based on the dynamic time warping (DTW) algorithm. Oquendo et al. [12] designed a magnetic induction motion tracking system and algorithm. The algorithm could automatically track the suture trajectory in order to assess the suture skills of trainees in pediatric laparoscopy. However, introducing these sensors, data gloves, and other extra tools [13] dramatically reduces training efficiency and increases the burden and cost of surgical skill assessment. Additionally, the software-based motion tracking system has been used to assess surgical proficiency. However, these methods have poor tracking accuracy [14,15]. Overall, despite the motion tracking system’s effectiveness in evaluating surgical competence according to the aforementioned assessment techniques, it is still challenging to integrate it into RAMIS training and assessment due to various problems, such as low efficiency and poor accuracy. Therefore, research on a RAMIS-appropriate assessment technique that is actually effective and accurate is urgently needed.

Based on the above needs, the automatic assessment of surgical skills using deep learning neural networks has become a hot research topic. The application of deep neural networks needs to be based on the datasets. Thus, many scholars have studied the RAMIS surgical skill assessment dataset. Rivas-Blanco et al. [16] explored the dataset that could be used to automate surgical robotic tasks, surgical skill assessment, and gesture recognition. In addition, the JIGSAWS [17] is one of the most widely available datasets for technical skill assessment in surgical robots. These large amounts of data can promote the development of surgical robot skill assessment towards automation. Kitaguchi et al. [18] proposed a deep learning method based on a convolutional neural network (CNN). It can achieve the high-precision automatic recognition of surgical actions with an accuracy rate of 91.9%. The long short-term memory (LSTM) model [19] and a symmetric dilated convolutional neural network model, SD-Net [20], have also been used for the automatic assessment of surgical skills. Nguyen et al. [21] described an automated assessment system using a CNN-LSTM neural network model and IMU sensors. This model performed classification and regression tasks for kinematic data in the JIGSAWS, achieving over 95% accuracy. Wang et al. [22] proposed an analytical deep learning framework for surgical training skill assessment based on sensor data and a CNN, implementing deep convolutional neural networks to map multivariate time series kinematics data to individual skill levels. Although their research achieved promising results, the experiments were based on existing datasets or sensor data. This was valuable for laboratory research, but it is still a long way from being practically applied to RAMIS. Our aim is therefore to develop a broadly applicable, scalable evaluation method that can be easily integrated into surgical robots.

In RAMIS, the endoscope can provide visual field information, which we believe can play a role in surgical skill assessment. No additional hardware is needed, which satisfies the demands of practical applications, if vision can be utilized in place of sensors to acquire signals. The motion signals are used as the input features of neural networks in RAMIS skill assessment [23]. Traditional kinematic data are no longer absolutely superior to visual data in surgical skill assessment [24]. Funke et al. [25] achieved a nearly 100% classification accuracy using 3D visual features. Evaluation methods based on 3D visual features tend to outperform 2D methods, but they have limited utility and are not suitable for RAMIS training. To help integrate automated skill assessment into surgical training practice, our proposed solution, therefore, relies on 2D visual features. Ming et al. [26] obtained over 70% accuracy when using 2D videos in surgical skill assessment; these videos represented the motion dynamics via improved dense trajectory (IDT) features and space temporal interest points (STIP). Lajkó et al. [27] demonstrated the potential application of optical flow in skill assessment using 2D vision during RAMIS and achieved an assessment accuracy of over 80%. The accuracy of 2D vision is not as good as that of 3D vision, but it has lower training costs and can be more efficiently applied to the automatic skill assessment in RAMIS. Therefore, this paper studies an intuitive and efficient assessment method using endoscopic 2D visual motion signals during RAMIS.

Based on the above problems associated with surgical skill assessment in RAMIS, this study proposed a new automated surgical skill assessment framework based on visual motion tracking technology; in addition, a deep neural network model that can be applied to real-time stage identification and online assessment is proposed. The new method utilizes a KCF algorithm [28] that can realize the motion tracking of the SIT. It establishes key motion signal features in the video. Meanwhile, the method employs a ResNet [29] model. It uses the visual motion signals as input in order to improve the classification efficiency of surgical skills and realize the efficient assessment of surgical skills. In addition, this method effectively considers the advantages of visual efficiency and the accuracy of motion signals, improving the assessment accuracy of surgical skills. Finally, the JIGSAWS is used to corroborate the effectiveness of the proposed method. The result shows that the classification of this method is better than that of other models. In this paper, a practical framework is provided for the automatic online assessment of objective skills in RAMIS.

To sum up, the innovations and contributions of this paper are as follows:

- A novel end-to-end analytical framework with visual tracking and deep learning is created for skill assessment based on the high-level analysis of surgical motion.
- Visual technology is used to replace traditional sensors in order to obtain motion signals in RAMIS.
- The proposed model is verified using the JIGSAWS dataset and the exploration of validation schemes applicable to the development of surgical skills assessment in RAMIS.

## 2. Materials and Methods

**Figure 1:**
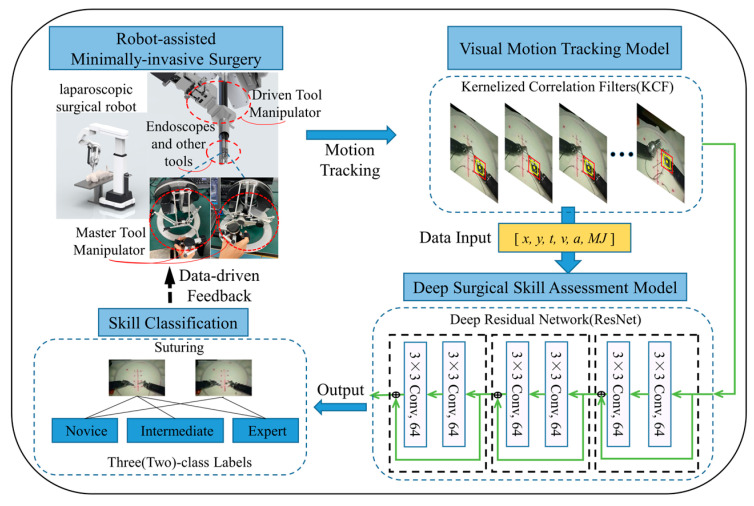
A framework for RAMIS based on a visual motion tracking and deep learning neural network.

The surgical skill assessment framework based on visual tracking and deep learning in RAMIS is shown in Figure 1. The endoscope at the end of the surgical robot was used to provide visual information, and the required motion signals of the SIT were recorded by the KCF, which is a multivariate time series (MTS), including [*x*, *y*, *t*, *v*, *a*, *MJ*] (Section 3.2.2). The recorded MTS was input into ResNet for classification. This outputs a discriminative assessment of surgical skills through a deep learning architecture, and the operator is then given the results. This chapter introduces the principles of the relevant models in detail.

### 2.1. KCF

The core part of most current trackers is the classifier, whose task is distinguishing the goals from the surroundings. In this study, the tracking model needed to accurately identify the SIT and capture their movements from the surroundings. The SIT moves at a relatively high speed when doctors perform surgical tasks, which is a great challenge for the tracking models.

The KCF is a high-speed and accurate motion-tracking algorithm, which has proven to be a very accurate tracking tool [30]. It is a kernel-based ridge regression classifier [31] that uses the cyclic matrix gained by cyclic displacement to collect positive and negative samples. The matrix operation is transformed into the point multiplication of the elements by using the diagonalization property of the cyclic matrix in the Fourier domain. The efficiency of calculation is improved. Meanwhile, the multi-channel histogram of oriented gradient (HOG) replaces the single-channel gray features and extends to multi-channel linear space to achieve higher robustness and accuracy.

**Figure 2:**
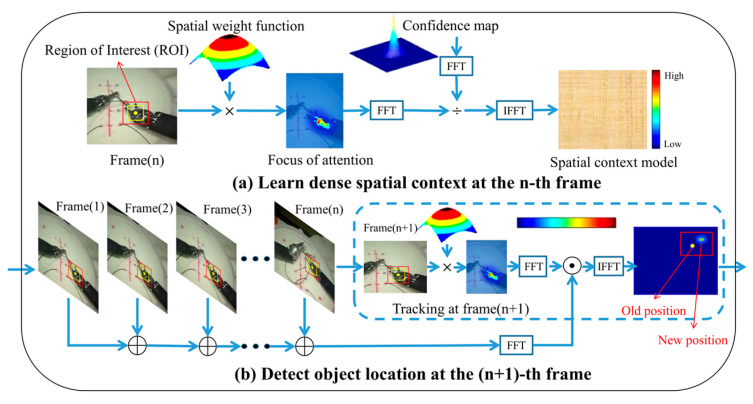
The basic framework of the kernelized correlation filter algorithm in this study: (**a**) is the training stage, and (**b**) is the detection stage. FFT is the Fast Fourier Transform, and IFFT is the inverse FFT.

As shown in Figure 2, the KCF mainly includes two stages, training and detection. In this study, the spatio-temporal context model [32] was used to learn about this framework. In the training stage, the features of the target region were extracted. Then, the kernel function was used to calculate the generation vector of the kernel matrix of the current regional features.

The KCF uses the multi-channel HOG features, which need to add vectors of different channel features. Taking the Gaussian kernel function as an example, Equation (1) is defined as follows:

(1) $$k^{xx^{\prime }}=exp(-\frac{1}{\sigma^{2}}({\left\|x\right\|}^{2}+{\left\|x^{\prime }\right\|}^{2}-2f^{-1}(\sum_{c}{\hat{x}}_{c}^{*}⊗{\hat{x}}_{c}^{\prime })))$$

where x is each sample in the circular matrix X, $f^{-1}$ is the inverse Fourier transform, $x^{*}$ is the complex conjugate of x, ${\hat{x}}^{*}$ is the discrete Fourier transform of $x^{*}$, and $k^{xx}$ is the first-row element of kernel function $k=C\left(k^{xx}\right)$.

Then, the filter template’s size is obtained using the kernel matrix and the ideal Gaussian output response. In the calculation, the kernel matrix is a cyclic matrix. Because of the large amount of data in the image, the kernel function can be diagonalized in the frequency domain to speed up the algorithm’s calculation. The kernelized ridge regression classifier weights are shown in Equation (2):

(2) $$\hat{\alpha }=\frac{\hat{y}}{{\hat{k}}^{xx}+\lambda }$$

where y is the output expectation and λ is the regularization coefficient of the filter template.

In the detection stage, the features of the candidate regions are first extracted, and then the current regional features are calculated using the kernel function. The rapid detection is shown in Equation (3):

(3) $$\hat{f}\left(z\right)={\hat{k}}^{xz}⊗\hat{\alpha }$$

The ideal regression expectation is the Gaussian, and the more like the tracking result of the previous frame it is, the greater the chance it is the tracking result of this frame. The center point in the next frame is more likely to be around the yellow point (inside the yellow box) in the region of interest (ROI), so the ideal regression is more likely to be in the center than around in Figure 2. The box’s position has changed, showing that the SIT has moved.

### 2.2. ResNet

**Figure 3:**
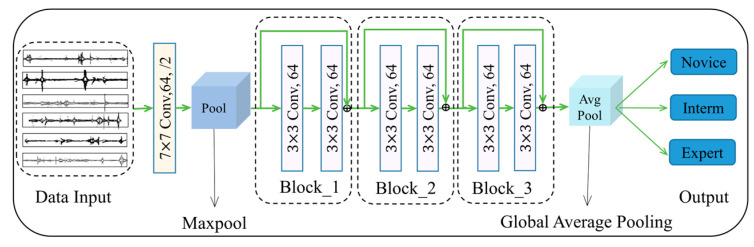
A 34-layer neural network structure with three residual building blocks.

The ResNet is mainly used for classification tasks [29]. The so-called skip connection is used to solve the degradation problem in ResNet. Essentially, it directly connects the shallow network to the deep one and can create a deeper one without losing performance. Even in a smaller network, it is also a reliable method. The overall network structure of the ResNet classification model in this study is shown in Figure 3. The features are fed into a convolution layer, followed by three residual building blocks. Finally, the results of classification are output. It should be emphasized that the model is selected after repeated tests during training and validation.

**Figure 4:**
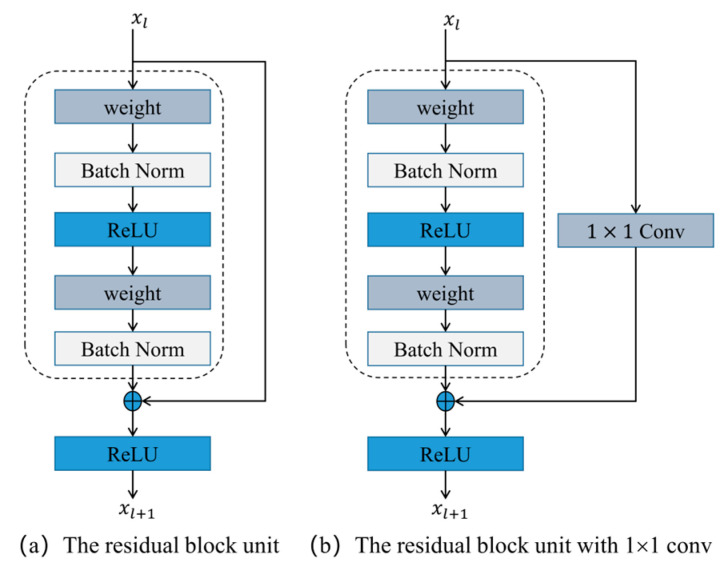
The residual building block in ResNet.

The ResNet is composed of a series of residual building blocks. A block model is shown in Figure 4, and it can be expressed as Equation (4):

(4) $$x_{l+1}=x_{l}+F\left(x_{l},W_{l}\right)$$

The residual building blocks contain two mappings: (1) the identity mapping, represented by $h(x_{l})$, which is the right curve in Figure 4a; and (2) the residual mapping. Residual mapping refers to the $F\left(x_{l},W_{l}\right)$ and generally consists of two or three convolutions, which is the left part in Figure 4a. In the convolution network, the number of feature maps in $x_{l}$ and $x_{l+1}$ may be different, and then the 1×1 Conv is needed to increase or reduce the dimension, which is shown in Figure 4b. The weight corresponds to 3×3 Conv,64, as shown in Figure 3. It can be expressed as Equation (5):

(5) $$x_{l+1}=h(x_{l})+F\left(x_{l},W_{l}\right)$$

where $h\left(x_{l}\right)=W_{l}^{\prime }x$ and $W_{l}^{\prime }x$ is the 1×1 conv.

## 3. Experimental and Results

### 3.1. Dataset

**Figure 5:**
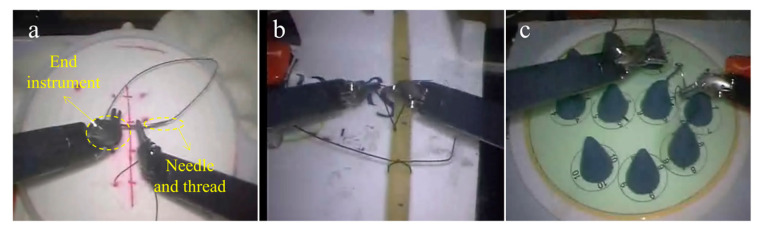
Three basic surgical tasks in the JIGSAWS. (**a**) suturing, (**b**) knot-tying, and (**c**) needle-passing.

We used the video collection in the JIGSAWS to simulate the manipulation motion of the surgical robot. The JHU-ISI Gesture and Skill Assessment Working Set (JIGSAWS) [33] was produced by Johns Hopkins University and Intuitive Surgery [34]. The JIGSAWS contains kinematic, video, and gesture data in three basic surgical tasks (suturing, knot-tying, and needle-passing). In the meantime, the JIGSAWS [35] contains a global rating score (GRS) that is determined using the upgraded Objective Structured Assessment of Technical Skills. Eight participants (B, C, D, E, F, G, H, and I), ranging from novices to experts at three levels, provided the data. As shown in Figure 5, the participants performed each task five times by controlling the da Vinci surgical robot. These three tasks are standard parts of the surgical skills training curriculum [17]. Two skill labels are recorded in the JIGSAWS: (1) The self-proclaimed skill label, which is based on surgical robot practice time. The experts reported more than 100 h, the intermediates reported between 10 and 100 h, and the novices reported less than 10 h; and (2) the labels based on the GRS (scores range from 6 to 30). This was performed manually by experienced surgeons. The higher the score, the higher the skill level. This study compared the skill levels, based on the GRS, to the self-proclaimed skill level, which was used as the true label for the trial.

This study focused on the suturing videos because it has a longer execution time and more complex actions in the JIGSAWS. Only the twenty-four suturing videos selected to ensure the same quantity of input from the novices, intermediates, and experts were used as the experimental object. These videos were recorded at a 30 Hz sampling frequency. Table 1 shows more details. It should be noted that the other two tasks used the same experimental methods in this study, and that we did not repeat them.

### 3.2. Experimental Setup

#### 3.2.1. Process of Visual Motion Tracking

**Figure 6:**
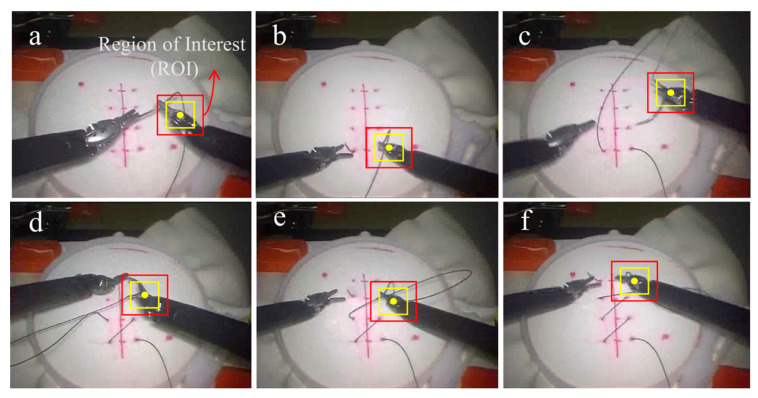
The process of suturing is shown from (**a**–**f**).

A tracking program was designed based on the KCF and ran in python. This program was used to automatically identify and track the ROI of the visible part of the SIT in the 2D continuous video frames and record the key motion signals. The quality of the surgical operation in RAMIS was presented by assessing the motion mode of the SIT. Such tracking methods have also been used to study the differences in physician hand movements during routine surgery [36,37]. The center pixel position of the ROI in each frame (every thirtieth of a second) in the videos was identified and tracked. Then, the position coordinates (*x*, *y*) and their running time (*t*) were automatically recorded. The KCF can overcome some short-time accidents, such as the instrument being blocked and covering the other, and motion mutation. However, the ROI position sometimes needs to be corrected, so we set the ROI so that it could be manually selected. As shown in Figure 6, the red box is the ROI selected manually. The center point in the next frame is more likely to be around the yellow point (inside the yellow box) in the ROI. The minor differences in the box’s size and position were ignored as long as the instrument was included.

**Figure 7:**
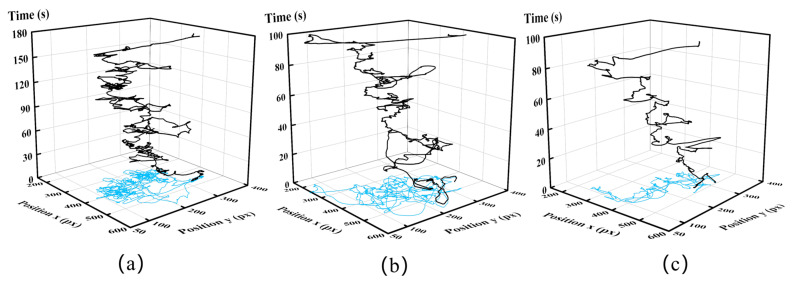
The SIT’s trajectory in a group of suturing. The blue part is the projection in the X–Y plane. (**a**) is from novices, (**b**) is from intermediates, and (**c**) is from experts.

The trajectory of the SIT is shown in Figure 7. The light blue part is the course projection in the X–Y plane. The length of the trajectory is 10,105 px, 8078.4 px, and 4317.5 px, respectively, which can be calculated by $d_{n+1}$ in Table 2. The trajectory curve of the novice is the most complicated, and the expert is the smoothest in the same suturing task. The novice has more redundant actions, thus taking 80 s more than the experts and intermediates to complete this suture task. Consequently, the distinction in the suturing skill of different operators can be seen clearly from the trajectory curve.

#### 3.2.2. Key Motion Futures

The tracking record for the position of the SIT can quantify the instantaneous displacement, velocity, acceleration, velocity curvature, and motion jerk [38]. In this study, the key motion features in Table 2 were recorded as the input of the ResNet in order to assess the surgical skills. Motion data were captured and saved into CSV files on the PC according to the surgical tasks and the expertise level of users via software implemented in Python. Some features were obtained by calculating the difference by code.

**Figure 8:**
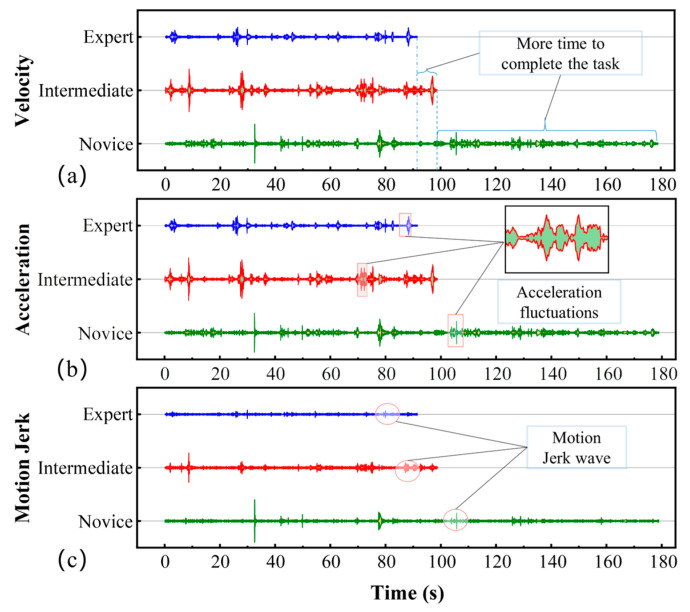
The signal graph of the motion of operators with three levels of expertise during suturing. (**a**) is the velocity and time, (**b**) is the acceleration and time, and (**c**) is the motion jerk and time.

The SIT’s velocity, acceleration, and motion jerk curves are shown in Figure 8 as a quantitative performance of speed–stationarity–smoothness. These key signals are important features used to measure surgical skills [38]. It can be seen that the three levels of operations show a linear trend. In addition, the swings of the curves are different, reflecting the distinctions among the actions of the three levels of operators. Compared to another two groups of operators, the curve of the experts has less swing and fewer abnormal data, which shows the smoother suturing and the higher quality of the expert.

#### 3.2.3. Implementation Details of Classification

This study’s assessment of surgical skills is formalized as a supervised classification problem. The input of the ResNet is the whole MTS of the kinematics of the end effector in the surgical robot, which is recorded by the KCF tracking model, including [*x*, *y*, *t*, *v*, *a*, *MJ*]. Each feature represents a dimension of the ResNet input vector. The length of each input vector data depends on the time of motion. This is accomplished by using the benchmark’s sliding window preprocessing method, which was implemented by Anh et al. [19]. The same padding is used in most places, to maintain the dimensions of the output.

(6) $$output\_width=\frac{W-F_{w}+2P}{S_{w}}+1$$

(7) $$output\_height=\frac{H-F_{h}+2P}{S_{h}}+1$$

where *W* and *H* are the width and height of the input, respectively, *S* is the stride length, *F* is the filter dimensions and *P* is the padding size (i.e., the number of rows or columns to be padded). In the case of the same padding, the following stands:

(8) $$output\_width=ceil(\frac{H}{S_{h}})$$

(9) $$output\_width=ceil(\frac{W}{S_{w}})$$

The output is a predicted label representing the corresponding professional level of the trainees, which can be encoded as 0: novice, 1: intermediate, and 2: expert. The hyper-parameters are selected empirically with a learning rate of 0.001 and a batch size of 24, and are trained in a maximum of 100 epochs. To implement this network topology, the ResNet is trained from scratch without any pre-training model. It runs based on Python, using the Keras library and TensorFlow on a computer with an Intel Core i5-10400F processor with 2.90 GHz and 16 GB RAM. To ensure that the results are more objective and accurate, as chosen by Anh et al. [19], each method is run five times for each generated input file. Within each run, five trials use the leave-one-super-trial-out (LOSO) cross-validation method, and the mean accuracy is calculated.

#### 3.2.4. Modeling Performance Measures

In this study, four common indexes [39,40] were applied to evaluate the performance of the classification model:

- accuracy, the ratio between the number of samples correctly classified and the total number of samples;

(10) $$accuracy=\frac{T_{p}+T_{n}}{T_{p}+F_{p}+F_{n}+T_{n}}$$

- precision, the ratio between the correct positive predictions and the total positive results predicted by the classifier;

(11) $$precision=\frac{T_{p}}{T_{p}+F_{p}}$$

- recall, the ratio between the positive predictions and the total positive results in the ground truth;

(12) $$recall=\frac{T_{p}}{T_{p}+F_{n}}$$

- F1-score, a weighted harmonic average between precision and recall.

(13) $$F1-score=\frac{2\times \left(recall\times precision\right)}{recsll+precision}$$

where $T_{p}$ and $F_{p}$ are the numbers of true positives and false positives, respectively, and $T_{n}$ and $F_{n}$ are the numbers of true negatives and false negatives for a specific class, respectively.

### 3.3. Results

**Figure 9:**
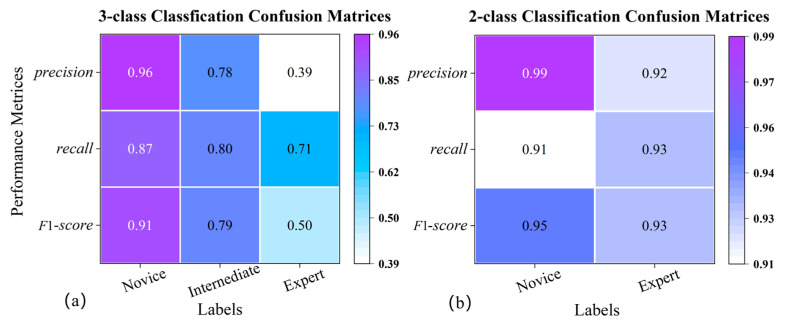
The confusion matrix for two and three classifications. (**a**) is the three-class result; (**b**) is the two-class result. The element value and color denote the probability of predicting skill labels, where the skill labels are self-proclaimed.

In this study, the proposed endoscopic visual motion tracking technology and deep learning neural network-based framework for automatically assessing surgical skills in RAMIS were validated using the JIGSAWS. Figure 9 shows the confusion matrix of the classification results. Figure 9a shows the complete three classifications, and Figure 9b uses the results of two classifications without the intermediates. Specifically, when the suturing task is classified into two and three classifications, respectively, the model’s accuracy is 92.04% and 84.80%. The performance of fewer class classifications is naturally better than more class classifications, but the reason why the gap is so significant is worth analyzing and discussing (Section 4). Among these three performance indicators, the three-class accuracy is fairly poor. However, the assessment of the novice group is more accurate, reaching 96%. For the experts group, the worst assessment classification accuracy is only 39%. The results are 3% and 53% higher than those of the three classifications when only labeled as novice and expert.

The results of this study can be compared to the most advanced classifications in Table 3. These studies used the JIGSAWS as a visual input source and performed experiments under the LOSO scheme. As can be observed, the new model generated results that were reasonably accurate, thus demonstrating the feasibility of the skill assessment method for RAMIS proposed in this study. 

**Figure 10:**
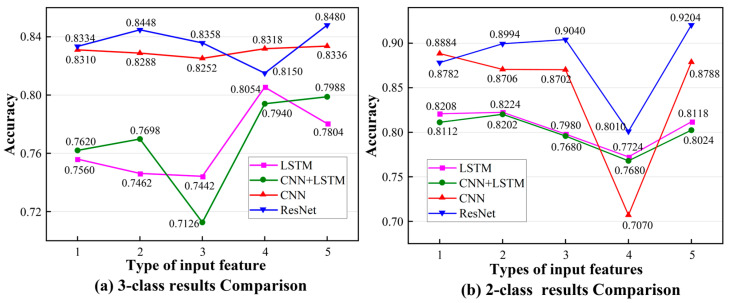
The comparison of classification results for different networks and different input features.

We performed a different set of experiments on LSTM, CNN, and CNN + LSTM in accordance with the same experimental settings and parameter configuration; this was in order to better support the ResNet. In Figure 10, the abscissa is the input features in the neural network, and the specific parameters are shown in Table 4. Firstly, the ResNet performs the best in the four neural networks when there are 5 input features. The accuracy of the ResNet in the case of three classes is 1.44%, 4.92%, and 6.76% higher than that of other networks. The accuracy of the ResNet in the case of two classes is 4.16%, 10.86%, and 11.8% higher than that of other networks. Secondly, the influence of the input features on the results is based on trajectory data. With the increase in input data, the classification accuracy is higher. However, in Figure 10b, the classification accuracy of the four input features decreased significantly, with a maximum reduction of 13.16% (ResNet). It can be seen that the trajectory data have a significant impact on the results of the two classifications. The problem of accuracy is discussed in Section 4. Only these four important motion features can be recorded due to the limitations of the present technology. If more features can be collected, the classification accuracy can be higher and the feedback on the results of the skill assessment might likewise be more precise.

Due to the network topology of the jump connection, the ResNet not only has higher classification accuracy, but is also competitive in terms of its computational efficiency, with the feedback of classification provided within 3 to 5 s, as shown in Table 5. Therefore, compared to other networks in this study, the ResNet is more appropriate for the framework of surgical skill assessment.

## 4. Discussion

### 4.1. Performance of the Framework

The proposed surgical skill assessment framework has been effectively validated using the JIGSAWS. The new model’s accuracy is 92.04% and 84.80% in the case of two and three classifications. It is proven that the new method can effectively and accurately assess the quality of surgical operation and skill level in RAMIS. However, it is worth mentioning that the intermediates and experts are prone to misclassification in the case of three classifications, and only 78% and 39% accuracy is achieved. These problems also appeared in the studies of Funk et al. [25], Anh et al. [19], and Lefor et al. [33]. To figure this out, we discussed the motion data gained via the KCF and the dataset.

### 4.2. Motion Features Assessment

**Figure 11:**
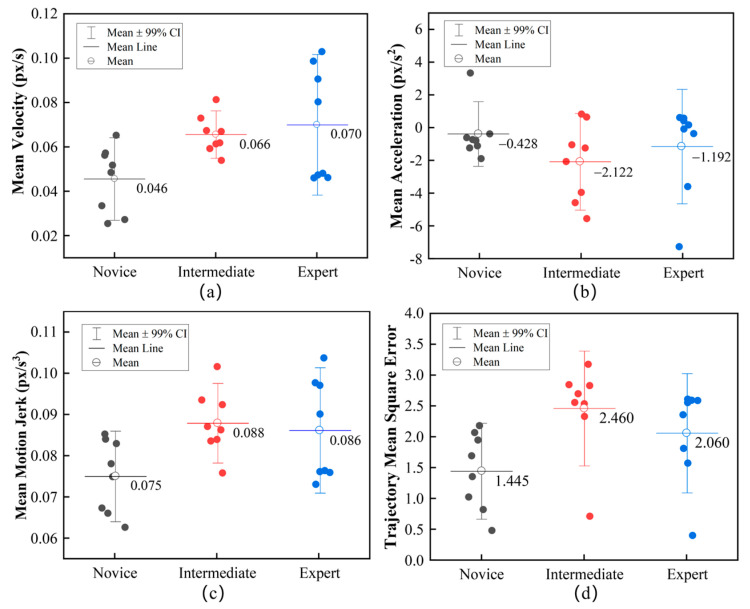
The motion features of the three-level performance in suturing. (**a**) is the mean velocity, (**b**) is the mean acceleration, (**c**) is the mean motion jerk, and (**d**) is the mean square error of trajectory.

In this paper, the motion features of the SIT are analyzed using the results recorded by the KCF algorithm. Figure 11 shows the mean value of the three motion features distributed within a 99% confidence interval (CI). In Figure 11a, the operating speed of experts and intermediates is close and only differs by 0.004 px/s. As shown in Figure 11b, the intermediates achieved the maximum acceleration. Despite this, they did not take the least amount of time, which means that many motion mutations of the SIT occur during movement. Figure 11c also shows that similar motion jerks occurred in both intermediates and experts, and only differed by 0.002 px/s^3^; however, the novices performed best. The presence of deviating points in the graph may be due to the misclassification of the dataset itself. The mean square error of trajectory (*S*) is an aggregate index that reflects how far a sample x and y deviate from the mean of all the samples. S shows a strong correlation with the ability to the instruments to perform an operation in the suturing task. Figure 11d shows the deviation degree of the trajectory points relative to the trajectory center. The larger *S* means exploratory or ineffective movement. Interestingly, what is reflected in Figure 11c,d is that novices have the best results. Because novices are often cautious when performing due to inexperience, the same action will take more time and lead to more detailed actions. In Equation (10), owing to the more significant number of sampling points n at the same length (compared with the other two levels), the minor difference between the continuous *x* and *y* results in a smaller *S*. With the mean square deviation and motion jerk, it is hard to distinguish the detail in the skills accurately. Therefore, the insignificant difference in the motions between the experts and intermediates means that the neural network cannot distinguish these two levels well.

(14) $$S=\sqrt{\frac{1}{n}\sum_{i=1}^{n}\left[{\left(x_{i}-\overset{-}{x}\right)}^{2}+{\left(y_{i}-\overset{-}{y}\right)}^{2}\right]}$$

where $x_{i}$ and $y_{i}$ are the two-dimensional coordinate values of the trajectory; $\overset{-}{x}$ and $\overset{-}{y}$ are the mean values of $x_{i}$ and $y_{i}$; and n is the number of sampling points.

### 4.3. Dataset Assessment

**Figure 12:**
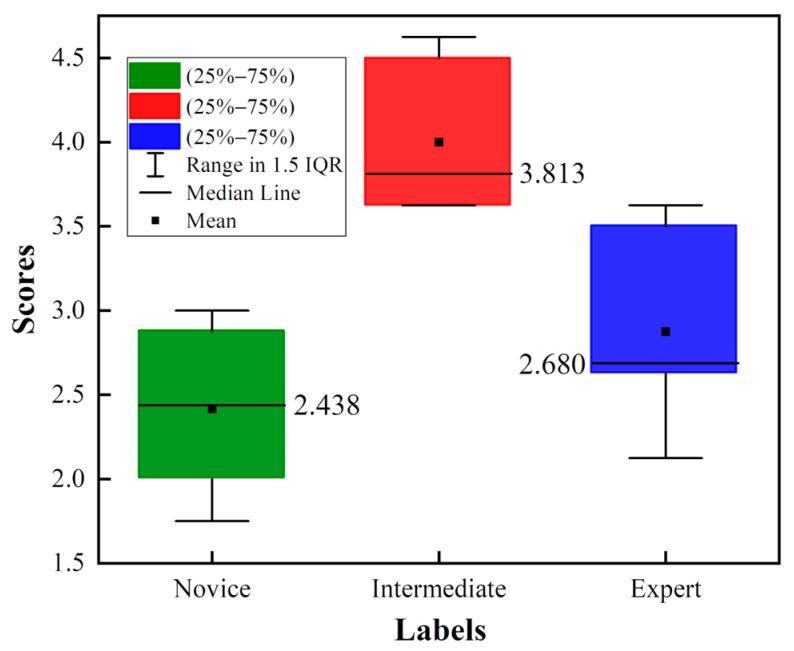
The box plot of the GRS of three skills.

The GRS in the JIGSAWS contains six scales scored from 1 to 5, including (1) respect for tissue, (2) suture handling, (3) time and motion, (4) flow of operation, (5) overall performance, and (6) quality of final product. Figure 12 shows the distribution of the GRS, thus reflecting the performance of the operation. The interquartile range (IQR) measures the degree of dispersion in the box plot. As can be seen, intermediates perform best overall, obtaining the highest composite score with a median of 3.813, followed by experts and novices. This means that there mismatch is between the GRS and the self-proclaimed skill labels. Therefore, the GRS in the JIGSAWS does not accurately distinguish the three levels of surgical operation skill.

The true labels of this article are the self-assessed skill ratings, which are based on the subjects’ practice duration. Naturally, one’s skill level tends to improve as one accumulates more practice time. However, each case is different. The dataset only labels subjects based on a training period of 10 h or 100 h, which is obviously insufficient to reflect the true situation. Therefore, as shown in Figure 12, the performance of intermediaries in the GRS skill assessment is generally better than that of the experts. There is an obvious conflict between the GRS and the self-assessed skill levels. As a result, the proposed classifier also made errors when distinguishing between intermediaries and experts. However, after removing intermediaries, the classification accuracy significantly improved. This indicates that the proposed classifier is still useful for training, and that the misclassification is caused by the incorrect labeling of the dataset itself. Therefore, the more accurate the labels, the better the performance of the assessment framework is.

### 4.4. Limitations and Future Research

The development of the RAMIS has promoted great research in objective skill assessment methods [41]. The current work has made some progress, but there are still some limitations to practicing online skill assessment when using this new model. First, this study has shown the potential use of the KCF in RAMIS skill assessment, proving that visual solutions may replace kinematics [42]. However, the accuracy of motion tracking cannot reach 100% accuracy during surgery due to the complex working environment and occlusion problems. Second, supervised deep learning classification accuracy depends mainly on labeled samples. This study focuses on the videos of the JIGSAWS, which lacks strict essential fact labels for skill levels. The self-proclaimed skill is labeled according to the operation time. It is not easy to judge whether it is true or accurate. In addition, skill labels are annotated according to predefined GRS score thresholds in GRS-based labels, but there is no universally accepted threshold. Thus, a more precise labeling method and more professional and in-depth surgeon knowledge may improve the skill assessment accuracy [43,44]. This paper uses the JIGSAWS dataset to conduct experiments to verify the proposed method. Although the experimental results are feasible, we must point out that the dataset we used is still too small. The final conclusion is only based on the suturing task in the JIGSAWS dataset, and more general conclusions need more datasets to support them. In addition, there is a lack of a clear definition of the intermediate between experts and novices, so the progressive assessment of more precise grades is currently not possible. Finally, the black box feature of the deep learning model further limits the interpretability of autonomous learning representations.

This work proposes a new and feasible method, rather than finding the best one. More advanced neural networks will be used in this framework in further studies. Endoscopic vision technology will be deeply studied in order to solve occlusion problems and obtain depth information effectively. The motion tracking technology in the three-dimensional space will be explored to further improve the accuracy of skill assessment based on endoscopic visual motion tracking technology. In addition, the deep topology, parameter settings, and improvement strategy of the deep learning neural network will be optimized in detail in order to better process the data of the motion time series and further improve the performance of online assessment.

## 5. Conclusions

Efficient and accurate skill assessment in RAMIS is essential in order to ensure patient safety. This study proposes a novel evaluation framework based on endoscopic visual motion tracking technology and deep learning. The new approach replaces traditional sensors with vision technology, innovatively combining vision and kinematics. The method uses the KCF to track and obtain two-dimensional motion signals based on endoscopic vision, such as the trajectory, velocity, and acceleration of the SIT. ResNet is then used for the automatic and accurate classification and analysis of surgical skills, and the results are compared with state-of-the-art research in the field. Finally, the reasons for some classification errors are discussed, and the limitations of this study are pointed out.

The contributions of this study are as follows: (1) The provision of an efficient and accurate framework for skill assessment in RAMIS, with classification accuracies of 84.80% and 92.04%, which can accurately provide feedback on online assessment results. (2) The simplification of the access process using the classification technology framework based on endoscopic vision and a neural network, and the realization of the feedback results within 3 to 5 s, thereby improving the efficiency of the assessment of surgical skills. (3) The automatic completion of the whole process of surgical skill assessment using the proposed method without employing additional tools other than the endoscope, so that it is more valuable for application.

In conclusion, the aim of this study was to propose a method for assessing surgical skills that combines vision and kinematics. The new method effectively considers the advantages of vision and kinematics in the assessment of surgical skills, achieving a higher level of two-dimensional visual assessment. It can be easily integrated and applied to the system in RAMIS. Real-time and accurate feedback can be obtained during personalized surgery, improving surgeon training efficiency and ensuring surgical quality and safety.

## Figures and Tables

**Table 1:** The needed details of the suturing tasks in this experiment.

Self-Proclaimed Skill Labels	Name	Number of Videos	Time (s)	The GRS
Novice	B, G, H, I	8	172.5 ± 58.3	14.5 ± 2.9
Intermediate	C, F	8	90.8 ± 15.1	24.0 ± 3.8
Expert	D, E	8	83 ± 13.3	17.3 ± 2.5

Some values shown are the mean ± standard deviation.

**Table 2:** The specific key motion feature parameters.

Symbol	Description	Formula
$t_{n}$	The time recorded at frame *n*	/
$x_{n}$	Position x coordinate at frame *n*	/
$y_{n}$	Position y coordinate at frame *n*	/
$d_{n+1}$	Distance moved between consecutive frames	$\sqrt{{\left(x_{n+1}-x_{n}\right)}^{2}+{\left(y_{n+1}-y_{n}\right)}^{2}}$
v	The mean velocity of the ROI in consecutive frames	$\sqrt{{\left(\frac{dx}{dt}\right)}^{2}+{\left(\frac{dy}{dt}\right)}^{2}}$
a	Mean acceleration of the ROI in consecutive frames	$\frac{dv}{dt}$
*MJ*	A parameter based on the cubic derivative of displacement with time, which refers to the change in the motion acceleration of the ROI used to study motion smoothness	$\sqrt{{\left(\frac{d^{3}x}{dt^{3}}\right)}^{2}+{\left(\frac{d^{3}y}{dt^{3}}\right)}^{2}}$

**Table 3:** The results of this study are compared to research reporting on the latest technology.

Author (Year)	Method	Suture
Ming et al. (2021) [26]	STIP	79.29%
Ming et al. (2021) [26]	IDT	76.79%
Lajkó G et al. (2021) [27]	CNN	80.72%
Lajkó G et al. (2021) [27]	CNN + LSTM	81.58%
Lajkó G et al. (2021) [27]	ResNet	81.89%
Current Study	KCF + ResNet	84.80%

**Table 4:** The meaning of types of input features.

Number	Input Features
1	[x,y,t]
2	[x,y,t,v]
3	[x,y,t,v,a]
4	[v,a,MJ]
5	[x,y,t,v,a,MJ]

**Table 5:** The computational processing time of different neural networks.

Input Features	Method	Time
[x,y,t,v,a,MJ]	CNN	1~3 s
	ResNet	3~5 s
	CNN + LSTM	24~48 s
	LSTM	16~68 s

## Data Availability

Publicly available datasets were analyzed in this study. The KCF algorithm source code can be found here: https://github.com/uoip/KCFpy, accessed date: 25 October 2022. The related neural network algorithm source code can be found here: https://github.com/SimonNgj/compssa, accessed date: 25 October 2022.
